# Overground Walking Decreases Alpha Activity and Entrains Eye Movements in Humans

**DOI:** 10.3389/fnhum.2020.561755

**Published:** 2020-12-22

**Authors:** Liyu Cao, Xinyu Chen, Barbara F. Haendel

**Affiliations:** Department of Psychology (III), Julius-Maximilians-Universität Würzburg, Würzburg, Germany

**Keywords:** mobile EEG, alpha oscillations, blinks, saccades, locomotion, pupil size, walking phase, motor entrainment

## Abstract

Experiments in animal models have shown that running increases neuronal activity in early visual areas in light as well as in darkness. This suggests that visual processing is influenced by locomotion independent of visual input. Combining mobile electroencephalography, motion- and eye-tracking, we investigated the influence of overground free walking on cortical alpha activity (~10 Hz) and eye movements in healthy humans. Alpha activity has been considered a valuable marker of inhibition of sensory processing and shown to negatively correlate with neuronal firing rates. We found that walking led to a decrease in alpha activity over occipital cortex compared to standing. This decrease was present during walking in darkness as well as during light. Importantly, eye movements could not explain the change in alpha activity. Nevertheless, we found that walking and eye related movements were linked. While the blink rate increased with increasing walking speed independent of light or darkness, saccade rate was only significantly linked to walking speed in the light. Pupil size, on the other hand, was larger during darkness than during light, but only showed a modulation by walking in darkness. Analyzing the effect of walking with respect to the stride cycle, we further found that blinks and saccades preferentially occurred during the double support phase of walking. Alpha power, as shown previously, was lower during the swing phase than during the double support phase. We however could exclude the possibility that the alpha modulation was introduced by a walking movement induced change in electrode impedance. Overall, our work indicates that the human visual system is influenced by the current locomotion state of the body. This influence affects eye movement pattern as well as neuronal activity in sensory areas and might form part of an implicit strategy to optimally extract sensory information during locomotion.

## Introduction

Exciting findings from electrophysiological research in the animal model suggest that an increased walking speed results in enhanced neural activity in the visual domain. The firing rates of neurons in the primary visual cortex of mice, a cortical area responsible for processing basic visual input, are modulated depending on the running speed even in complete darkness (Niell and Stryker, 2010; Keller et al., 2012; Ayaz et al., 2013; Polack et al., 2013; Saleem et al., 2013; Fu et al., 2014; Lee et al., 2014; Dadarlat and Stryker, 2017; Clancy et al., 2019). For review, please see Busse et al. (2017) and Händel and Scholvinck (2017). This modulation goes beyond the influence of arousal (Vinck et al., 2015). Similar effects have been reported in invertebrates (Chiappe et al., 2010; Maimon et al., 2010; Weir et al., 2014). While there is behavioral and electrophysiological work indicating that movements and cognitive processes such as memory, attention and perception are linked in humans (Mcmorris and Graydon, 2000; Gramann et al., 2010; Schmidt-Kassow et al., 2013; De Sanctis et al., 2014; De Vos et al., 2014; Kranczioch et al., 2014; Lin et al., 2014; Wascher et al., 2014; Bullock et al., 2015, 2017; Labonté-Lemoyne et al., 2015; Conradi et al., 2016), work showing an influence of locomotion on visual cortical activity as shown in animals is sparse in humans. In this respect, two recent studies showed that walking can lead to increased surround suppression (Benjamin et al., 2018) and peripheral visual information processing (Cao and Händel, 2019) in humans. Both studies provided evidence that locomotion can also lead to a change in visual processing of a stimulus, as has been shown in animals.

In human non-invasive electrophysiology, one possibility to look at ongoing activity is the power analysis of sustained oscillatory activity. Alpha activity, as a marker of inhibitory activity (Klimesch, 1999; Klimesch et al., 2007; Händel et al., 2011), has been found to be closely related to the firing rates of single neurons (Haegens et al., 2011). A reduction in alpha power during walking could therefore mark an increased activity in humans comparably to that found in animals. Indeed there have been previous reports of decreased oscillatory power in the alpha band (~10 Hz) during movements (e.g., walking) and navigation (Ehinger et al., 2014; Lin et al., 2014; Scanlon et al., 2017; Cao and Händel, 2019). Additionally, there are reports of alpha and beta oscillatory power (mainly over sensorimotor cortex) being modulated by the phase of walking (see e.g., Gwin et al., 2011; Wagner et al., 2014; Roeder et al., 2018). A typical finding is that alpha power is lower during the swing phase compared to the double support phase of walking. However, it is not clear if the phase modulation of brain oscillations during walking is contributed by an electrode impedance change. It is possible that a specific walking phase (e.g., the heel strike) may lead to a change of the electrode impedance due to a small turbulence introduced by the movement.

When participants are allowed to freely move without any restrictions, as in the current study, it is very important to keep in mind that movements influence each other. Body movements have been shown to interact on various scales. For example, if the head is free to move, an increased saccade rate can be found (Kowler, 1991). Whereas, if the head is fixed, very small saccades referred to as microsaccades will increase in rate and/or amplitude (Collewijn and Kowler, 2008). Walking and eye-related movements also seem to be tightly linked. A recent study has shown a delicate coordination between gaze and gait cycle during natural overground walking (Matthis et al., 2018). Earlier work additionally suggests a temporal relationship between foot and eye movements by showing that saccades during walking are most likely made in the double support phase just before the toe-off (Hollands and Marple-Horvat, 2001).

In the current study, we ask if the alpha power decrease during walking is independent of visual input (e.g., in the dark) and thereby comparable to the animal findings. The walking phase modulation of alpha power is further investigated by measuring the electrode impedance during a full stride cycle. Additionally, the effect of walking and walking phase on eye movements (e.g., saccades) is investigated. This is an interesting question by itself but also contributes to the interpretation of the changed brain activity during walking as changed eye movements may also lead to a changed brain activity.

## Materials and Methods

### Participants

Thirty healthy participants (20 females; mean age = 29.2; SD = 8.0) were recruited from a local participant pool for the main experiment, and 18 healthy participants (10 females; mean age = 31.5; SD = 4.7) were recruited for the electrode impedance measurement during walking. All participants gave written informed consent prior to the study and received monetary compensation after the study. The study was approved by the local ethics committee (Department of Psychology, University Würzburg) and was conducted in accordance with the Declaration of Helsinki and the European data protection law (GDPR).

### Task and Procedure

In the speed condition, participants were standing still (speed = 0), slow walking (low speed) or normal walking (normal speed) (i.e., 3 levels; main experiment). Participants chose their own comfortable walking speed with the constraint that they should walk slower in the slow walking as compared to the normal walking. Participants were engaged with normal overground walking instead of treadmill walking as commonly seen in previous walking studies (e.g., Lin et al., 2014). Each speed was conducted in two lighting levels: in the dark or in the light. Therefore, this is a 2 (lighting condition: light vs. dark) by 3 (speed condition: standing still, slowing walking, and normal walking) within-subjects design. Each of the 6 levels was tested in a 76.5-s session, and the order was randomized. Given the prominence of alpha activity in EEG data, a 76.5-s recording session should be more than enough to achieve a fair assessment of the activity strength. A 1-s tone was delivered in the beginning and at the end of each session signaling the session start/end. There was a break of about 2 min between each session. A 3D-printed black plastic blindfold, consisting of a goggle like structure (fitted around eye tracking glasses, see below for details) with a front cover, together with an opaque canvas wrapped around the participant's head created a maximally dark environment. Only a few participants reported seeing very dim, constant beams in the created dark environment. In the light level, the front cover of the blindfold was removed. The average field of view measured from two participants in a pilot study was 110° (horizontal) and 50° (vertical). Participants were told to keep their eyes open in all testing sessions. Prior to the current task, participants had completed another unrelated EEG study involving a visual detection task as described elsewhere (Cao and Händel, 2019). A controlled saccade test and some questionnaire data were collected after the task. The experiment was conducted in a large activity hall (about 30 × 50 m; wooden floor) of the university gym. In all sessions in which walking was required, participants stayed within an area of about 100 m^2^ in the center of the activity hall. The experimenter was always present, quietly monitoring the walking path. A warning was given when participants walked too close to the wall. In total, nine participants received on average three warnings when walking in the dark.

For the impedance measurement, another 18 participants were tested (different cohort from the main experiment). The task was similar to the normal walking session in the main experiment (in the light). Each participant was tested for 3 min in two separate testing sessions: one session with the electrode impedance measured throughout the 3-min testing, and the other without. The testing was conducted in a smaller room (about 5 × 6 m).

### Data Recording

**Figure 1:**
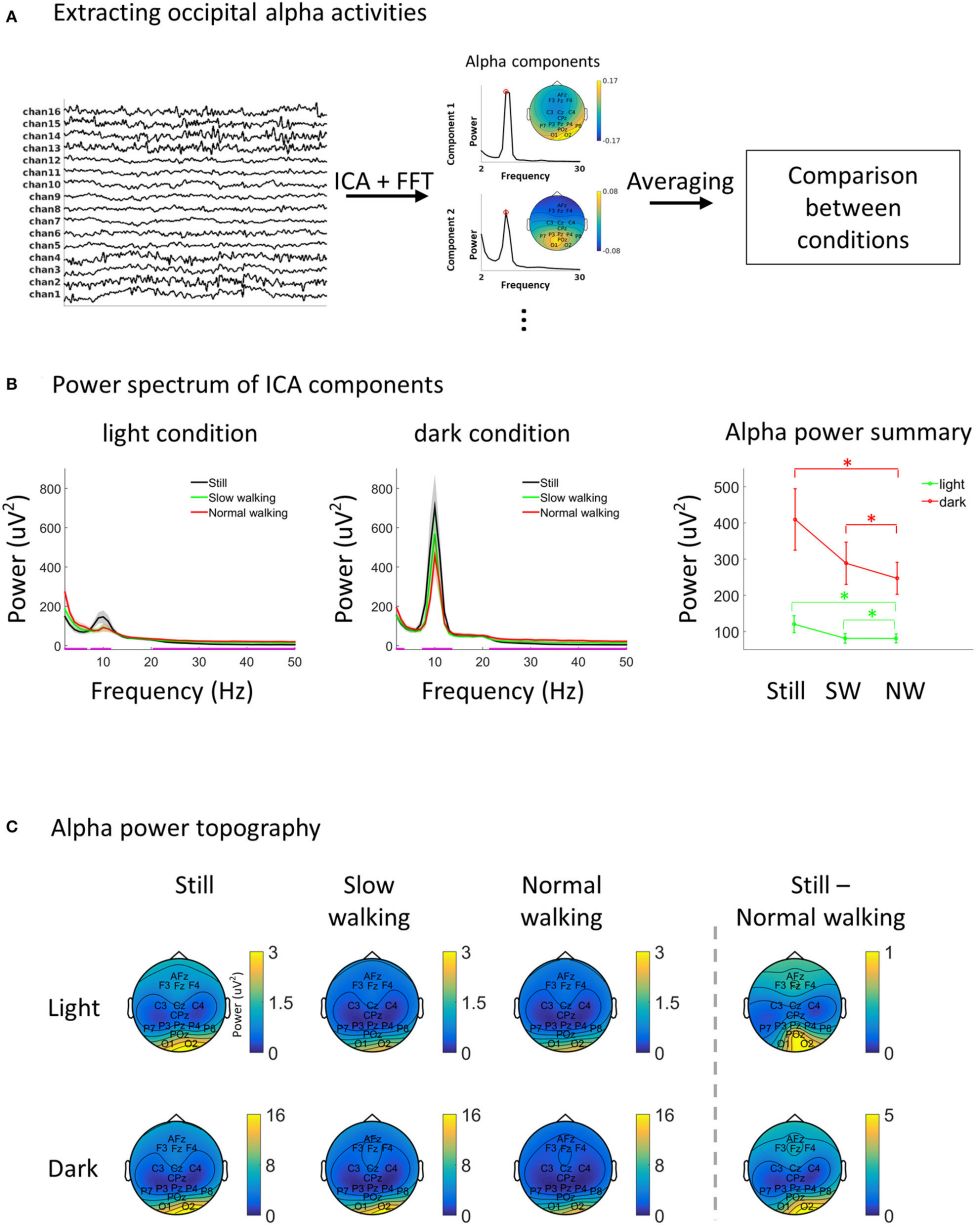
Alpha decreases during walking. **(A)** A schematic illustration of the EEG data analysis approach. Preprocessed data were subjected to independent component analysis and the ICA components with high alpha power from the occipital area were identified automatically. The selected ICA components were then averaged within each testing session for further comparison. **(B)** EEG power spectra from alpha ICA components in each condition (left and middle). The magenta line on the abscissa marks the frequency bands showing significant differences between speed levels (FDR adjustment for multiple comparison) separately for the light and dark. One the right, the alpha power in each condition was plotting separately for better visualization. Asterisks indicate *p* < 0.05. Shading and vertical lines indicate ±1 standard error. *N* = 26. **(C)** The topography of alpha power in each condition. The strongest activation (column 1–3) and the most prominent difference between standing still and normal walking (column 4) were found in the occipital area.

EEG data were collected using a Smarting mobile EEG system (mBrainTrain LLC, Serbia), which has 24 recording channels with a sampling rate of 500 Hz. We used 6 channels for EOG (electrooculogram) recording (for each eye: one below and one above the eye, one to the outer canthus) and 18 channels for EEG recording (with one electrode on each earlobe for possible re-referencing; see Figure 1C for EEG channel distribution). A common mode sense active electrode placed between Fz and Cz was used for online reference. The EEG signal amplifier and data transmitter are integrated into a little box (82 × 51 × 12 mm; 60 grams) which is attached to the back of the EEG cap. Data transmission is achieved via bluetooth. Motion data (velocity and acceleration; sampling rate: 120 Hz) were collected using a Perception Neuron system (https://neuronmocap.com/products/perception_neuron; Noitom Ltd., China). Three-dimension velocity and three-dimension acceleration data were collected from three sensors: one attached to each foot ankle (a few centimeters above the lateral malleolus) and the third one attached to the participant's back (at the waist level). The motion sensors were firmly attached on the top the participants' clothes. The pupil size was measured, only in the main experiment, with mobile eye-tracking glasses (SMI-ETG, SensoMotoric Instruments GmbH, Germany), with a sampling rate of 120 Hz. The glasses were worn under the blindfold during the testing. Triggers for recording session start/end were generated with the software Lab Streaming Layer (https://github.com/sccn/labstreaminglayer), which was also used for collecting and synchronizing other streams of data (EEG, motion data and pupil size data). A Dell laptop (model: Latitude E7440) was used for running the experimental program. During the experiment, participants carried the laptop in a rucksack.

### Data Analysis

All the data analysis detailed below was completed using the Fieldtrip toolbox (Oostenveld et al., 2011) and in-house scripts running on Matlab (The MathWorks Inc., USA).

### Alpha Power

For each 76.5-s testing session, the EEG data were re-referenced to the grand average (excluding EOG channels and the two earlobe channels, i.e., 16 channels were used for analysis) before a low-pass filter at 100 Hz (a windowed sinc finite-impulse-response filter with Kaiser windowing was used for all the filtering processes unless otherwise stated) and a high-pass filter at 1 Hz were applied. The filtered data from six testing sessions were then combined into one data structure and reduced to 10 dimensions with principal component analysis, which was followed by an independent component analysis (ICA) using the “runica” (Infomax) approach (Delorme and Makeig, 2004). The power spectrum of each ICA component was obtained using Welch's method (1 s time window, 50% overlap, 1 Hz resolution). ICA components were selected for further analysis if an alpha peak was found in the power spectrum and if the component topography has higher loading on occipital sensors than other sensor. Specifically, a component was selected if all the following requirements were met: 1. There were local peaks from 6 to 14 Hz in the component power spectrum; 2. The width of the local peak, which was defined as the frequency width between the two adjacent local minimums, should be at least 4 Hz (avoiding noisy transient peaks). If there were more than one local peak, the local peak with the largest width was taken (also for step 3); 3. The power at the local peak frequency should be at least 3 times higher than the mean power between 20 and 50 Hz (avoiding components with a broad band high power); 4. In the ICA topography, the maximum absolute weight from sensors in the occipital area (O1, O2, and POz) was larger than the maximum absolute weight from all other sensors. Four participants were excluded as no components fulfilled the above requirements. On average, 2.4 components (SD = 0.8) were obtained from the remaining 26 participants. Each alpha component was partitioned back into the six testing sessions, and the average power spectrum of all components was calculated for each testing session. For alpha topography (Figure 1C), the selected components were projected back into sensor space and the alpha power was obtained for each sensor using Welch's method with the aforementioned parameters.

### Saccade Detection

Saccade detection was based on the so-called REOG (radial EOG) component, which is the difference between the mean of all six EOG channels and the Pz channel. REOG component was band-pass filtered between 20 and 90 Hz using a 6th order Butterworth filter and then Hilbert transformed to obtain the amplitude envelop. All data points where the amplitude value deviated from the mean by 2.5 standard deviations were considered saccade-related and were grouped into one saccade if they were <20 ms apart. The above saccade detection procedure was performed separately for each testing session. This method was shown to be able to detect saccades very reliably when there are no head movements (Keren et al., 2010). However, applying the same method to a free walking setting, one needs to keep in mind that the method actually detects a relative movement between the eyes and the head. When the eyes maintain fixation head movements may lead to an increase in the amplitude of REOG signal. The vestibulo-ocular reflex would be an example of such behavior. We want to point out that during our experiment there was no constraint on head or eye movements. We assume that most eye movements were part of the normal orienting movements that combine head and eye movements in addition to small corrective movements. A classical vestibulo-ocular reflex as introduced by unexpected head movements or head rotation during forced fixation did not occur during our experiment. In Supplementary Figure 1A, the saccadic spike potentials of detected saccades were shown, which seem quite comparable to those detected during head fixation.

### Blink Detection

Blinks were detected from the vertical EOG component, i.e., the amplitude difference between the EOG channels above and below eyes. The vertical EOG component was high-pass filtered at 0.2 Hz and low-pass filtered at 10 Hz. A blink was marked if the vertical component crossed an individually defined threshold. The threshold was determined based on the amplitude of EOG component from individual data through visual inspection (the testing condition information was not available to the researcher during inspection) and the same threshold was used for all testing sessions of the same participant. The mean threshold across participants was 50.8 μV (SD = 11.8 μV). Blinks with the ratio between amplitude standard deviation and mean amplitude smaller than 0.2 were excluded. Adjacent blink points within 100 ms were combined into one blink. Results of blink detection from both eyes were quite similar. Only results from the left eye were used.

### Pupil Size

Raw pupil size (radius) data were quite noisy. For the complete pupil size data series (i.e., all the six testing sessions including the break time between sessions), missing data points were first excluded before a low pass filtering at 0.5 Hz was performed. Data from two participants were excluded as proportions of missing data points were more than 99%. The missing data points were then filled using a linear interpolation method (matlab “fillmissing” function). The complete pupil size data were then grouped into each testing session and the average pupil size in each testing session was calculated (results were very similar between filled data and the data excluding missing points). For the remaining 28 participants, the three speed levels in the light had mean missing points of 6.7% (SD = 9.8%, standing still), 8.6% (SD = 11.2%, slow walking) and 9.2% (SD = 11.7%, normal walking), and in the dark had a mean of 15.3% (SD = 21.8%, standing still), 14.2% (SD = 17.4%, slow walking) and 18.8% (SD = 24.8%, normal walking). A 2 (lighting condition: light vs. dark) by 3 (speed condition: standing still, slow walking, and normal walking) within-subjects ANOVA (analysis of variance) comparing the proportion of missing data points gave only a significant main effect of lighting (F_(1,27)_ = 11.39, *p* < 0.001). The pupil size from the left eye was used (right eye data were similar to the left eye and produced statistical results not qualitatively different from the left eye in all related tests).

### Analyzing the Relationship Between Alpha Power and Eye-Related Movements

To analyse the possible relationship between alpha power and eye-related movements, alpha power in each 1 s epoch was obtained. The 76.5-s data in each session (i.e., the selected ICA components) were divided into epochs of 1-s with 0.5 s overlap between epochs (912 epochs in all testing sessions). The alpha power in each epoch was obtained using a fast Fourier transform after applying a Hamming window, which would give the same result of the mean alpha power in the whole 76.5 s as Welch's method did. Within each session, epochs with extreme alpha power were excluded using the MAD-median rule: let p be the alpha power in an epoch and P be the alpha power of all epochs within a testing session. If |p – median(P)| x 0.6745 > 2.24 × MAD-median (the median absolute deviation from the median), this epoch is an outlier (Wilcox and Rousselet, 2018). The epochs were grouped depending on the number of saccades/blinks detected or the average pupil size. Epochs with more than 5 saccades (5.5% of all epochs) or more than 1 blink (9.2% of all epochs) were excluded. On average, there were 146.77 epochs (SD = 55.89) with 0 saccade, 239.46 epochs (SD = 40.65) with 1 saccade, 251.77 epochs (SD = 36.46) with 2 saccades, 163.12 epochs (SD = 48.58) with 3 saccades, and 64.73 epochs (SD = 22.40) with 4 saccades in all testing sessions. There were 472.08 epochs (SD = 172.68) with 0 blink, and 355.77 epochs (SD = 115.90) with 1 blink. For each participant, the pupil size data were divided into five groups from lowest pupil size to highest pupil size with equal number of epochs within each group. Alpha power was then compared between different groups separately for saccade rate, blink rate, and pupil size.

### Walking Phase and Related Analyses

The walking phase information was estimated from the velocity data (see also **Figure 3A** for an illustration). Three-dimension velocity data were transformed to one-dimension speed data using the following formula:

$$\begin{array}{l} \text{speed}=\text{sqrt}\left({\left(\text{velocity\_x}\right)}^{\wedge }2+{\left(\text{velocity\_y}\right)}^{\wedge }2+{\left(\text{velocity\_z}\right)}^{\wedge }2\right), \end{array}$$

where velocity_x, velocity_y, and velocity_z indicate the velocity data in three orthogonal dimensions. For each testing session, the speed time series from both ankles were added up and the combined speed time series was low-pass filtered at 2 Hz (walking speed data from one participant was missing due to technical error). Local minimum speed points were identified from the combined speed time series as the start point of a stride cycle (note that the start point of a stride cycle was also the end point of the stride cycle before). The stride cycle, as obtained here, did not distinguish between left foot stride and right foot stride. In all related analysis, stride cycles (steps) with a particularly long duration (i.e., the duration between two consecutive local minimum speed points) were excluded using the MAD-median rule. The local maximum speed point between two local minimum speed points was also identified for plotting the average speed profile of stride cycles (**Figure 3B**). The average duration of stride cycles was 1.13 s (SD = 0.35 s) during slow walking in light, 0.62 s (SD = 0.08 s) during normal walking in light, 1.14 s (SD = 0.32 s) during slow walking in dark, and 0.66 s (SD = 0.10 s) during normal walking in dark. The average number of stride cycles was 68.4 (SD = 17.5) during slow walking in light, 120.6 (SD = 17.3) during normal walking in light, 65.5 (SD = 14.9) during slow walking in dark, and 108.4 (SD = 11.9) during normal walking in dark. For the analysis of possible modulatory effects from walking phase, the time period between two local minimum speed points (i.e., one stride cycle) was divided into 9 smaller equally spaced time periods, i.e., nine walking phases. Since the walking phase was defined based on the continuous walking speed data, we refer to a phase as low speed phase when the walking speed reaches a local minimum (i.e., in both ends of a stride cycle) and a phase as high speed phase when the walking speed is high (i.e., in the middle of a stride cycle). The low speed phase may correspond to the canonically defined double support phase when both feet are touching the ground. The high speed phase (especially phase 5 in the middle) would correspond to the canonically defined swing phase with one foot in the air. The average alpha power, saccade rate, blink rate, and the average pupil size were computed for each walking phase. The alpha power evolution over time was also calculated for each testing session. This is necessary for the analysis of the relationship between alpha power and walking phase. Each alpha ICA component was band-pass filtered (Hamming window) between 8 and 12 Hz. A Hilbert transform was applied to the filtered data so that a power envelope can be obtained for each component before averaging. Note that the alpha power obtained in this way was much larger in amplitude as compared to the alpha power obtained using Welch's method. Steps that with extreme alpha power were excluded using the aforementioned MAD-median rule.

### Electrode Impedance

The continuous impedance data can be calculated from the signal power at 125 Hz for each electrode with the Smarting mobile EEG system when the impedance measurement was switched on. The power at 125 Hz was obtained for each sampling point using a 200 ms window after applying a Hamming tapper. The error in the measured impedance is about 1.5 kohm, so the impedance may appear slightly below 0 when the connection between the scalp and the electrode is very good. The impedance from four electrodes showing very strong alpha activities (“O1,” “O2,” “P7,” “P8”) were averaged, and sorted into different walking phases before being compared (similar to the analysis of the relationship between walking phase and alpha power). Data from two participants were not included due to technical error during the recording, leaving 16 participants in the final results.

### Statistical Analyses

Within-subjects ANOVA, e.g., a 2 (lighting condition: light vs. dark) by 3 (speed condition: standing still, slow walking, and normal walking) within-subjects ANOVA comparing alpha power, and (two-tailed) paired *t* test were carried out for various comparisons detailed in the results section. Throughout the manuscript, all statistical comparisons were non-parametric, which was implemented through the randomization test (parametric statistics gave similar results in each of test reported in the whole manuscript). In the randomization test, the variables were randomly exchanged between conditions within each participant to obtain the distribution of the statistical variable (F value in ANOVA, mean difference between conditions in *t* test) under the null hypothesis, i.e., there are differences between conditions. The statistical variable obtained from the original data (no randomization) was then compared to the null distribution to derive a *p* value. The within-subjects ANOVA algorithm was implemented by Gladwin (Gladwin, 2020), and the paired *t* test was implemented by ourselves. Statistical results are reported as significant when the *p* value is smaller than 0.05, and corrections for multiple comparisons were mentioned in the text whenever they were performed.

## Results

### Walking State Modulates Alpha Power, Eye-Related Movements, and Pupil Size

The average walking speed as detected by a motion sensor attached to the back was 0.53 m/s (SD = 0.18) during slow walking in light, 1.12 m/s (SD = 0.17) during normal walking in light, 0.50 m/s (SD = 0.17) during slow walking in dark, and 0.96 m/s (SD = 0.16) during normal walking in dark. A 2 (lighting condition: light vs. dark) by 2 (speed condition: slow vs. normal walking) within-subjects ANOVA indicated that participants walked significantly faster during normal walking than during slow walking [F_(1,28)_ = 243.34, *p* < 0.001], and in light than in dark [F_(1,28)_ = 29.86, *p* < 0.001]. A significant interaction was also found [F_(1,28)_ = 16.48, *p* < 0.001], which was due to the fact that there was no significant difference in speed during slow walking between light and dark [t_(28)_ = 1.65, *p* = 0.11], but normal walking was significantly faster in light than in dark [t_(28)_ = 5.47, *p* < 0.001].

ICA components with strong alpha activity (8–12 Hz) were extracted (Figure 1A). Unsurprisingly, alpha activities from the extracted ICA components were very strong (Figure 1B). A one-way within-subjects ANOVA was first carried out to compare the power spectra between speed levels for each frequency between 2 and 50 Hz, separately for the light and the dark lighting levles (multiple comparisons were adjusted with false discovery rate, FDR) (Yekutieli and Benjamini, 1999). Significant differences in the alpha band were found in both lighting levels. We then specifically compared alpha power using a 2 (lighting condition: light vs. dark) by 3 (speed condition: standing still, slow walking, and normal walking) within-subjects ANOVA. The results showed significant main effects of lighting [F_(1, 25)_ = 16.84, *p* < 0.001] and speed [F_(2, 50)_ = 11.49, *p* < 0.001]. A significant interaction was also found [F_(2, 50)_ = 5.70, *p* < 0.001]. Alpha power was lower in the light (mean = 94.08; SD = 78.20) compared to the dark (mean = 315.05; SD = 309.42). In the light, alpha power was lower in both slow walking [mean = 81.07; SD = 67.18; t_(25)_ = −2.44, *p* = 0.01] and normal walking [mean = 80.92; SD = 62.66; t_(25)_ = −2.67, *p* = 0.003] compared to standing still (mean = 120.26; SD = 119.59), with no significant difference being found between slow walking and normal walking [t_(25)_ = 0.03, *p* = 0.99]. A similar pattern was found in the dark, with alpha power being lower in both slow walking [mean = 288.55; SD = 297.80; t_(25)_ = −4.03, *p* < 0.001] and normal walking [mean = 246.94; SD = 225.05; t_(25)_ = −3.13, *p* < 0.001] compared to standing still (mean = 409.67; SD = 432.69), and not significantly different between slow walking and normal walking [t_(25)_ = 1.36, *p* = 0.20]. The interaction effect indicates that the effect of alpha decrease during walking in the dark [within-subjects one-way ANOVA comparing between speed levels: F_(2, 50)_ = 9.45, *p* < 0.001] was stronger than in the light [within-subjects one-way ANOVA comparing between speed levels: F_(2,50)_ = 6.08, *p* = 0.004]. The topography of alpha power in each condition showed a strong activation in the occipital area (sensors O1 and O2), suggesting a visual origin of the alpha oscillations considered here (Figure 1C). The strongest alpha power difference between standing still and normal walking (still – normal walking) was also found in occipital sensors.

**Figure 2:**
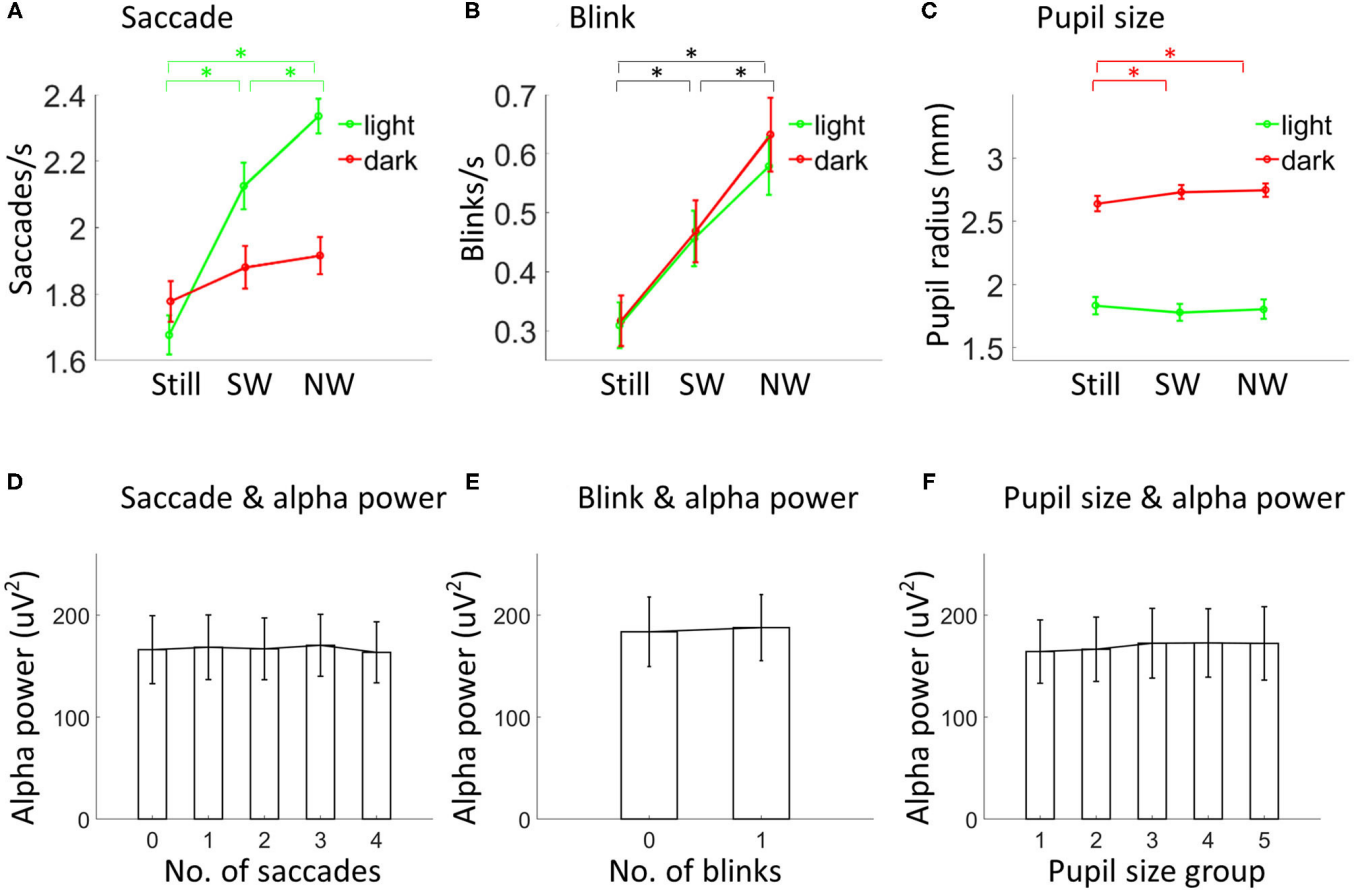
Eye-related movements, pupil size and alpha power. Saccade rate (**A**; *N* = 30), blink rate (**B**; *N* = 30) and pupil size (**C**; *N* = 28) depicted separately for each condition. Asterisks indicate *p* < 0.05. Black lines in **(B)** indicate comparisons in both light and dark. Alpha power was not modulated by the number of saccades (**D**; *N* = 26), blinks (**E**; *N* = 26), or the average pupil size (**F**; *N* = 24). In **(F)**, the pupil size increased from group 1 to group 5. Vertical lines indicate ±1 standard error. SW, slow walking; NW, normal walking.

The same 2 (lighting condition: light vs. dark) by 3 (speed condition: standing still, slow walking, and normal walking) within-subjects ANOVA was then performed to compare saccade rate, blink rate and pupil size. For saccade rate (see Supplementary Figure 1A for the saccadic spike potential for detected saccades), both main effects of lighting [F_(1, 29)_ = 11.23, *p* = 0.001] and speed [F_(2, 58)_ = 40.18, *p* < 0.001] were significant, including a significant interaction effect [F_(2, 58)_ = 17.13, *p* < 0.001] (Figure 2A). *Post-hoc* analysis for the interaction effect showed that saccade rate increased with speed in the light (slow walking vs. standing still: {t_(29)_ = 7.11, *p* < 0.001; normal walking vs. slow walking: [t_(29)_ = 3.58, *p* < 0.001]} but not in the dark (all comparisons between speed level pairs gave *p* values larger than 0.05). For blink rate, only the main effect of walking was significant [F_(2,58)_ = 31.52, *p* < 0.001], indicating that blink rate increased with speed {slow walking vs. standing still: [t_(29)_ = 3.75, *p* < 0.001]; normal walking vs. slow walking: [t_(29)_ = 7.42, *p* < 0.001]} (Figure 2B). For pupil size, the main effect of lighting [F_(1, 27)_ = 228.50, *p* < 0.001] and the interaction effect [F_(2, 54)_ = 3.51, *p* = 0.04] were significant (Figure 2C). *Post-hoc* analysis for the interaction effect indicates that, in the dark, pupil size increased from standing still to slow walking [t_(27)_ = 2.40, *p* = 0.02] and to normal walking [t_(27)_ = 2.22, *p* = 0.03], with no difference being found between normal and slow walking [t_(27)_ = 0.42, *p* = 0.69]. Whereas, in the light, no differences could be found between any speed level pairs (all *p* values were >0.27).

### No General Link Between Alpha Power and Eye-Related Movements

Since walking modulates both alpha power and eye movements, it is important to ask if alpha power and eye-related movements are related in the current dataset. To test this, the 76.5-s data in each testing session were divided into epochs of 1-s with 0.5 s overlap between epochs. For each epoch, alpha power, numbers of saccades and blinks, and the average pupil size were obtained. Alpha power was not modulated by the number of saccades [F_(4, 100)_ = 0.97, *p* = 0.44] or blinks [F_(1, 25)_ = 0.001, *p* = 0.98] for data collapsed over all testing sessions (Figures 2D,E). Pupil size data were sorted into five groups from smallest pupil size to largest pupil size. Again, alpha power was not found to be different between groups [F_(4, 92)_ = 0.72, *p* = 0.68; Figure 2F].

### Walking Phase Modulates Alpha Power

**Figure 3:**
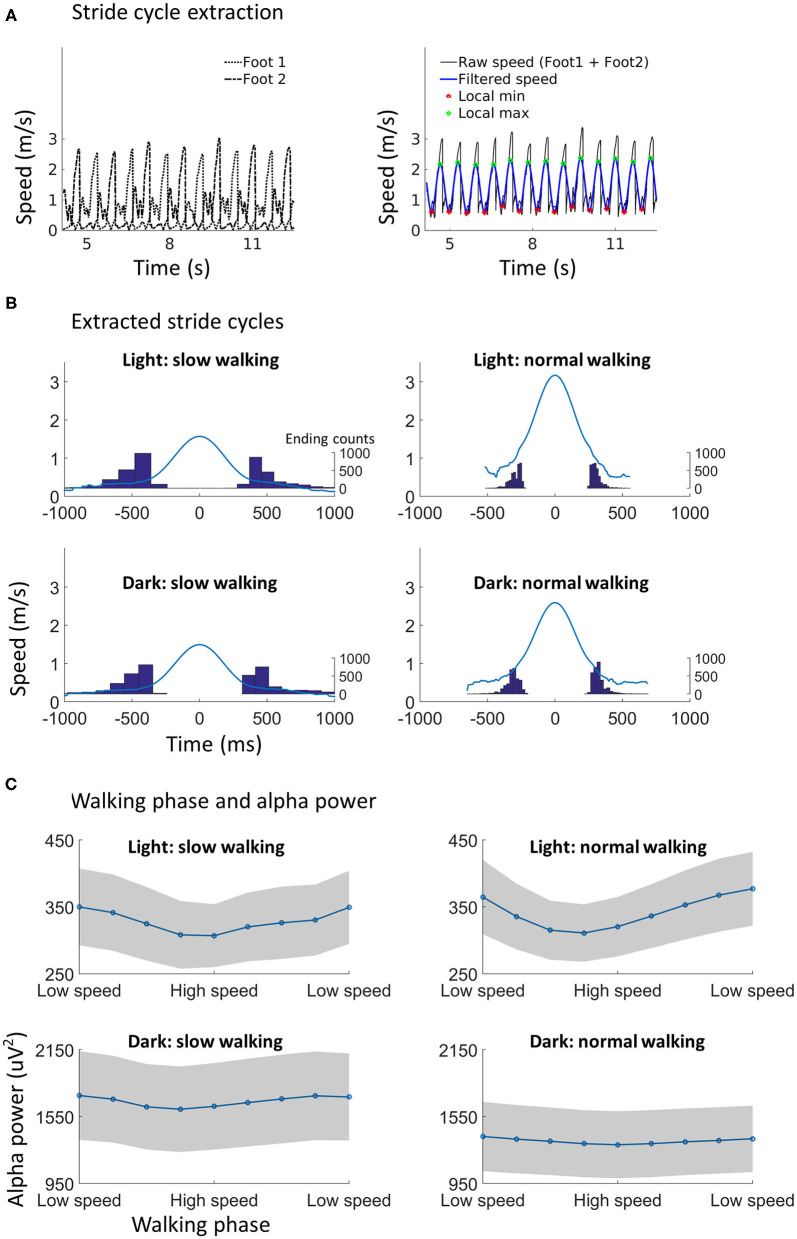
Walking phase modulates alpha power. **(A)** Illustration of stride cycle extraction. The walking speed from both ankles (left) were added up, and the combined raw walking speed time series data were filtered (right). The local minimum point and the local maximum point in the filtered data could then be found using the phase information. The local minimum point was used as the start/end point of a stride cycle. A subset of speed data during normal walking in the dark from one participant was used for the illustration. **(B)** The extracted stride cycles from all participants (*N* = 29) in each condition. The walking speed of each stride cycle was aligned to the local maximum point (as shown in **A**), which is time point 0 on the x-axis, for calculating the average speed profile (blue line). The distribution of the duration between the local minimum point and the local maximum point is shown with histograms, with scales shown on the right of each plot. **(C)** Alpha power in different walking phases. Each stride cycle (from a local minimum to the next local minimum) was divided into nine equal time windows. The low speed phase and high speed phase may roughly correspond to the canonically defined double support phase and swing phase, respectively. A clear modulation of alpha power by walking phase can be found. Shading indicates ±1 standard error. *N* = 25.

The walking phase was obtained based on the speed information from both ankles. Speeds from both ankles were added up and the local minimum speed point was detected as the start and the end point of a stride cycle (Figures 3A,B). Each stride cycle was then divided into nine equally long successive time periods, equivalent to nine walking phases, with the first and the last phase (low speed phase) roughly corresponding to the double support phase and the middle phase (high speed phase) corresponding to the swing phase. The average alpha power within each phase was computed. Any modulatory effect from walking phase should lead to different strength of alpha power in different phases, which was tested with a 2 (lighting condition: light vs. dark) by 2 (walking speed condition: slow vs. normal walking) by 9 (walking phase) within-subjects ANOVA.

Significant main effects were found for lighting [F_(1, 24)_ = 13.87, *p* < 0.001] and walking phase [F_(8, 192)_ = 6.68, *p* = 0.004]. The effect of lighting showed that alpha power was higher in the dark than in the light, which has been shown in the previous section. The effect of walking phase indicated that alpha power was lower in the low speed phase as compared to the high speed phase during a stride cycle (Figure 3C). No other effects from the three-way ANOVA were significant.

### Walking Phase Does Not Modulate Electrode Impedance

**Figure 4:**
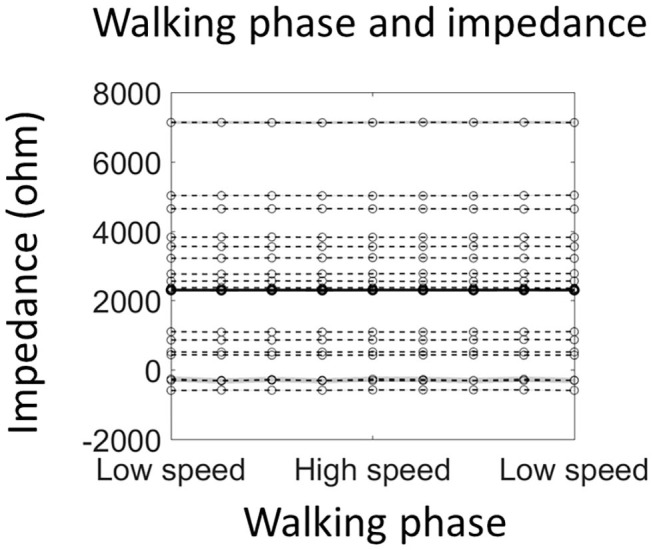
Walking phase does not modulate electrode impedance. The solid line shows the average impedance of all 16 participants, and the dashed line shows individual data (shading indicates ±1 standard error of measurements across all stride cycles). Note that negative impedance was due to the measurement error and indicated very good connection between the scalp and the electrode.

The electrode impedance was measured during normal walking with an additional 16 participants to test the possibility that the alpha power modulation by the walking phase, as reported in the last section, is due to an impedance change resulting from walking movements. However, the average impedance of four occipital sensors was not modulated by the walking phase [F_(8, 120)_ = 0.71, *p* = 0.80; Figure 4]. Importantly, a significant alpha power modulation by the walking phase could still be detected from the 4 sensors [F_(8, 120)_ = 4.72, *p* = 0.01; Supplementary Figure 2].

### Walking Phase Modulates Eye Movements

**Figure 5:**
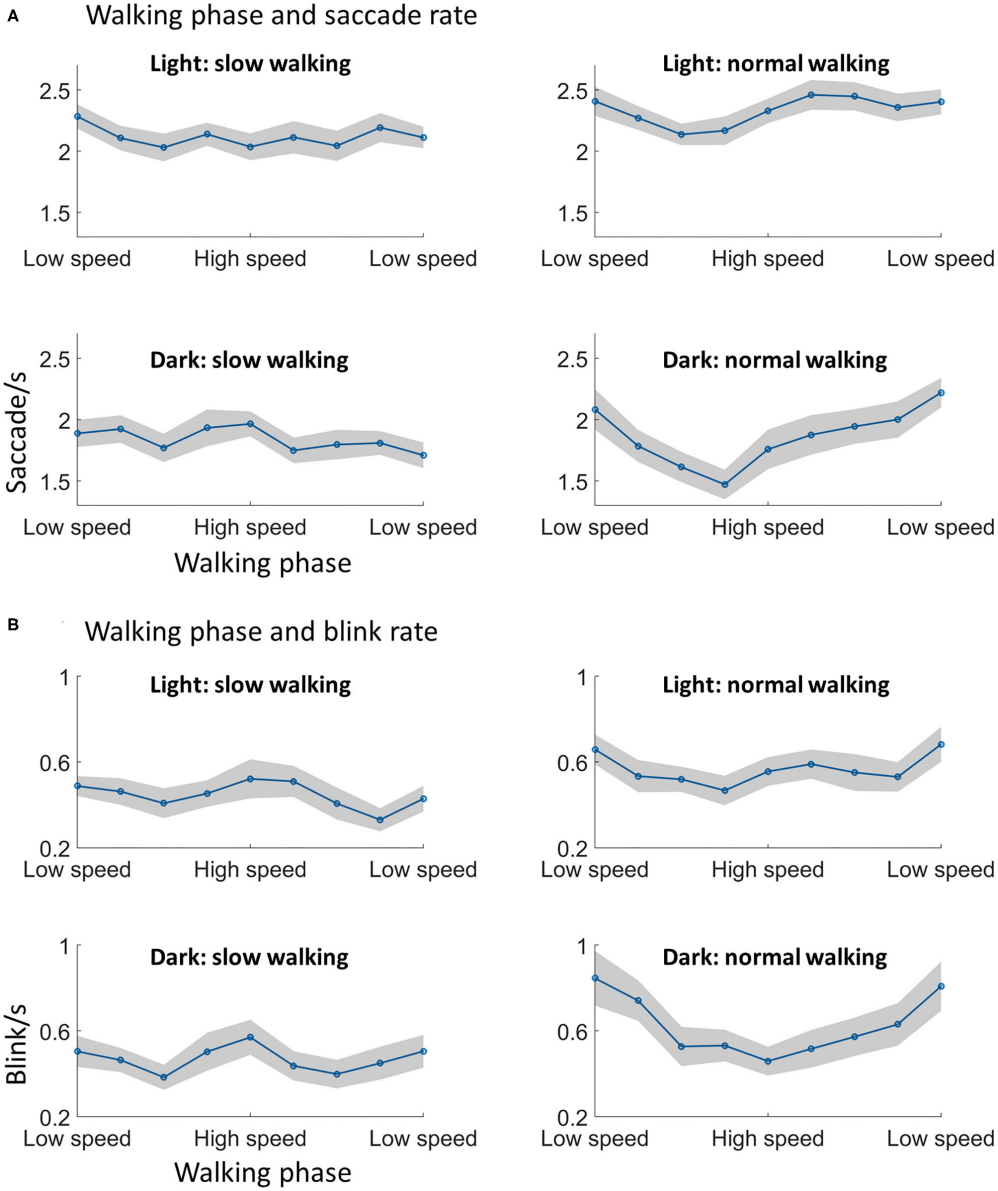
Walking phase modulates saccade and blink rate. **(A)** The saccade rate was lower in the high speed phase as compared to the low speed phase, which was only found in normal walking. **(B)** A similar modulation during normal walking was found with the blink rate, i.e., participants showed less blinks in the swing phase of walking. Shading indicates ±1 standard error. *N* = 29.

For saccade rate, significant main effects from the ANOVA were found for lighting [F_(1, 28)_ = 27.37, *p* < 0.001], walking speed [F_(1, 28)_ = 7.72, *p* = 0.01], and walking phase [F_(8, 224)_ = 2.39, *p* = 0.02] (Figure 5A). The effects of lighting and walking conform to previous results showing that saccade rates increase from dark to light, and from slow walking to normal walking. The effect of walking phase indicated that the saccade rate was different across walking phases. Furthermore, a significant interaction effect was found between walking speed and walking phase [F_(8, 224)_ = 2.76, *p* = 0.02]. *Post-hoc* analysis indicated that the saccade rate was modulated by walking phase during normal walking [F_(8, 224)_ = 3.40, *p* = 0.004; one-way within-subjects ANOVA], but not during slow walking F_(8, 224)_ = 0.97, *p* = 0.45; one-way within-subjects ANOVA. During normal walking, the saccade rate appeared to be lower during the high speed phase than during the low speed phase. No other effects from the three-way ANOVA were significant.

For blink rate, a similar walking phase modulation effect was observed. Significant main effects from the ANOVA were found for walking speed [F_(1, 28)_ = 37.38, *p* < 0.001] and walking phase [F_(8, 224)_ = 2.68, *p* = 0.02] (Figure 5B). The effect of walking speed showed that the blink rate increased with walking speed, as already showed in the previous analysis. The effect of walking phase indicated that the blink rate was different across walking phases. A significant interaction effect was also found between walking speed and walking phase [F_(8, 224)_ = 3.52, *p* = 0.01]. Similar to the pattern showed by the saccade rate, *post-hoc* analysis indicated that the blink rate was modulated by walking phase during normal walking [F_(8, 224)_ = 3.71, *p* = 0.004; one-way within-subjects ANOVA], but not during slow walking [F_(8, 224)_ = 1.81, *p* = 0.09; one-way within-subjects ANOVA]. During normal walking, the blink rate appeared to be lower during the high speed phase than during the low speed phase. No other effects from the three-way ANOVA were significant.

For pupil size, only a main effect of light was significant [F_(1, 26)_ = 200.32, *p* < 0.001] from the ANOVA, which indicated that the pupil size was smaller in the light than in the dark. See Supplementary Figure 3 for the pattern of pupil size across walking phases.

## Discussion

With the study at hand, we show that natural walking leads to lower occipital alpha power as well as an increased saccade and blink rate and a larger pupil size. The modulation of alpha power and blink rate by walking was observed when participants were walking in light as well as in dark, suggesting that the visual input during walking is not the driving factor. Walking phase was further found to modulate alpha power, and blink and saccade rate in both light and dark. We discuss these findings below in detail.

### Alpha Power Decrease Due to Walking (Walking State Modulation)

In both light and dark, alpha power considerably decreased during walking. Previous studies have reported such a modulation of alpha power by walking (Gwin et al., 2011; Ehinger et al., 2014; Lin et al., 2014; Scanlon et al., 2017; Cao and Händel, 2019). However, our results add important insights to this finding that help us to understand this prominent alpha modulation due to walking.

First, our results clearly indicated that alpha reduction during walking can be observed with a focus over occipital sensors, most likely reflecting a change of neural activity in the visual cortex. Previous studies on the alpha (or mu) modulation during walking focused on sensorimotor processing (Gwin et al., 2011; Severens et al., 2012; Lin et al., 2014; García-Cossio et al., 2015; Storzer et al., 2016; Scanlon et al., 2017). The current study provided a strong support for the existence of a similar modulation effect of walking on visual cortical activity in humans to the effect as demonstrated through single cell recordings in animals. A reduction in alpha activity during walking in humans, which would imply a disinhibition of cortical activity, is consistent with the finding of increased firing rates in the visual cortex of mice during locomotion (e.g., Saleem et al., 2013). Please find below a discussion on the validity of our alpha results with respect to localization. Second, we showed that the modulation of alpha power due to walking is independent of the visual input. In the dark, participants reported absolute darkness except a few participants, who reported a very faint and vague sensation of light, which entered through the connecting area between the shutter and the eye tracker. Third, our finding demonstrated such an effect in a task-free natural walking setting. This indicates that the necessity to focus on a specific task is not relevant to the alpha power modulation by walking. Fourth, we showed that saccades, blinks, and pupil size could not explain the alpha power modulation by walking. It should be noted that the data analysis in this part (Figures 2D–F) was not tailored for a proper investigation of the relationship between alpha power and eye movements/pupil size. Therefore, a lack of relationship does not necessarily mean no relationship (e.g., see alpha lateralization in the process of making a saccade; Belyusar et al., 2013). Rather, the results only indicate that the observed alpha power decrease during walking cannot be explained by changes in eye movements or pupil size during walking. Fifth, evaluating the change in signal to noise ratio, we saw an overall power increase in high and low frequencies during walking as compared to standing (Figure 1B; significant differences were indicated by the magenta line). However, the alpha modulation consisted of a decrease in the frequency limited alpha band. This is a clear indication, that the alpha change is not introduced by a walking induced change in noise. By additionally excluding order effects through an order randomization of all testing sessions, our finding therefore suggests a specific link between alpha power and walking.

What could be the functional reason for such a decrease in alpha power during walking compared to standing still? Previous studies have identified a sensorimotor mu/beta suppression during walking as compared to standing (Wagner et al., 2012, 2014). The modulated alpha activity during walking, as shown in the current study, was specifically picked up from the visual brain area (Figure 1C). In general, alpha activity, found in sensory areas, is often associated with attentional processes. Attending to a particular location or time point has been shown to lower the occipital alpha power which was further correlated with the improvement of visual perception (Van Dijk et al., 2008). Alpha power over visual cortex further reliably indicates the spatial focus of covert visual attention showing an increase in power for the unattended input (Worden et al., 2000; Fu et al., 2001; Yamagishi et al., 2003; Sauseng et al., 2005; Kelly et al., 2006; Thut et al., 2006). The preferred interpretation of alpha power picked up over sensory areas is therefore inhibition of sensory input processing (Klimesch, 1999; Klimesch et al., 2007; Händel et al., 2011). A functional interpretation of the observed decrease in alpha power during walking could therefore be linked to a change in the attentional state while walking. A recent study presented behavioral and neurophysiological evidence that walking shifts preferred visual processing toward the peripheral visual field (Cao and Händel, 2019). An overall reduction in alpha power during walking compared to standing still could therefore depict the reduced inhibition of the peripheral input.

Interestingly, alpha power decrease could be associated with an increase in neuronal activity investigating somatosensory areas in the monkey (Haegens et al., 2011). Interpreting our walking induced alpha power decrease in the light of the likely associated increase of neuronal activity, we believe that our findings are very well in line with recent animal work showing that activity in early visual cortex is increased by walking speed in complete darkness (Keller et al., 2012; Saleem et al., 2013; Erisken et al., 2014; Dipoppa et al., 2018). The significantly reduced alpha power in the light compared to the dark was further consistent with the idea that the alpha power analyzed in the current work indexed the neural processing of visual input (see also Cram et al., 1977; Ben-Simon et al., 2013). Taken together, these exciting findings point to a general, species-unspecific modulation of perceptual mechanisms due to movement.

### Walking State Modulates Eye-Related Movements and Pupil Size

In addition to changes in neuronal oscillatory power, walking had differential effects on eye related movements depending on the lighting level. The saccade rate increased with speed in the light but not in the dark. This is consistent with the idea of feedforward control of walking with visual information (Hollands and Marple-Horvat, 2001; Matthis et al., 2017). The increased saccade rate during walking in the light coincides with the increased processing demand of fast changing visual information to assist walking. No such processing demands of new visual information are required in the dark as no information is available. This might explain why the saccade rate did not increase when participants walked in the dark. Interestingly, the blink rate was also increased with speed. Visually demanding tasks, like a search task, are usually associated with a significantly decreased blink rate (Bauer et al., 1985; Cho et al., 2000). The finding, that the blink rate is equally affected by walking during darkness and light, further indicates that the modulation of blinking due to walking is independent of the visual input. We therefore assume that the increase in blink rate might be of the same nature as the link between blinking and speaking (Von Cramon and Schuri, 1980; Cruz et al., 2011) or blinking and button pressing (Van Dam and Van Ee, 2005; Cong et al., 2010) and rather based on a motor related interaction.

Pupil size increased while walking compared to standing in the dark, conforming to previous reports in mice (Erisken et al., 2014; Vinck et al., 2015). In the light, pupil size was expected to be modulated similarly (Benjamin et al., 2018), but for our data it showed no difference between speed levels in light. We suspect that a modulation could have been masked by changing luminance in the light. The testing room was non-uniformly lit (one side of the room was a bit brighter) which led to a change in luminance due to walking within this room. This would lead to an increase in variance of the measured pupil size, thereby masking moderate changes due to speed (Supplementary Figure 1B). Future studies on the pupil size during free walking should take the lighting condition into account. In the dark, the influence of luminance was absent.

### Walking Phase Related Modulation of Alpha Power

A walking phase related modulation in alpha power was found. Previous studies have shown a walking phase dependent power modulation in theta, alpha, beta, and gamma bands in a wide brain network including, posterior parietal, sensorimotor, and premotor areas (Gwin et al., 2011; Chéron et al., 2012; Severens et al., 2012; Seeber et al., 2014, 2015; Bradford et al., 2016; Artoni et al., 2017; Oliveira et al., 2017). The observation of walking phase dependent alpha power modulation in the current study is consistent with those reports. However, we note that the walking phase modulation of alpha power should be interpreted cautiously, as the level of artifacts in the EEG signal may be different in different walking phases. There are competing accounts with regards to the level of artifacts in the EEG signal during walking (Castermans et al., 2014; Nathan and Contreras-Vidal, 2015; Snyder et al., 2015), but it is feasible that the double support phase may have more artifacts as the heel-strike (foot landing on the ground) is in this phase (e.g., see an example of EEG noise assessment during walking from Bradford et al., 2016). Our findings give further evidence that the observed alpha power modulation is unlikely due to artifacts since the electrode impedance did not change over the stride cycle (Figure 4). In fact, the difference in impedance between different walking phases was so small (<100 ohm) that it cannot lead to any difference in the amplitude of measured signal (Ferree et al., 2001).

### Walking Phase Modulates Eye Movements

The result of walking phase dependent eye movements corroborates such a non-visual link for blinks and it additionally suggests that also saccades are not exclusively influenced by visual input. For saccades, a preference for the double support phase (just before the toe-off) has been reported previously while visual input was present (Hollands and Marple-Horvat, 2001). We show that besides the saccade rate, the blink rate was also modulated by the walking phase and that both rates increased during the double support phase compared to the swing phase. It is important to note that the walking related artifact cannot explain the pattern of saccade rate as modulated by the walking phase (saccades were detected based on the spectral power between 20 and 90 Hz from the REOG component). A 2 (lighting condition: light vs. dark) by 2 (walking speed condition: slow vs. normal walking) by 9 (walking phase) within-subjects ANOVA testing the amplitude of the saccadic spike potential did not give a significant effect of walking phase [F_(8, 224)_ = 0.87, *p* = 0.53] (Supplementary Figure 1C).

Considering the rhythmic nature of human bipedal walking, there could be a critical phase during which the execution of saccadic eye-movements is least disturbing for information acquisition. Due to saccadic inhibition, information processing is greatly reduced during the time of a saccade (Matin, 1974). During a blink, visual input is shut down due to eye lid closure as well as due to blink suppression (Volkmann et al., 1980). Since the double support phase, which includes heel-strikes, is also the moment when the visual system is most unstable due to the whole-body vibration imposed by heel-strikes, information acquisition might be suboptimal during this time point. Thus, a good strategy might be to make a blink/saccade during this double support phase so that it can be avoided in the swing phase when the visual system is relatively stable. Interestingly, the coupling between walking phase and preferred saccade as well as blink occurrence was also present in the dark. This makes the phase modulation effect specifically intriguing. Saccades and blinks do not have any effect on visual information in the dark. Nevertheless, they were still modulated by walking phase similarly to and even possibly slightly stronger than in the light. One possible explanation is that the preference of saccade occurrence during walking phase, once introduced by visual feedback, is now hard-coded and independent of vision. Therefore, it might be the case that the walking phase modulation effect on saccades and blinks is controlled by a low-level neural circuit and much easier to be observed in the dark compared to light, in which case the visual input from the environment may have an additional influence on saccades and blinks.

The reason why the phase modulation effects for eye related movements in normal walking but not in slow walking is not clear. One possibility might be that deliberate walking (i.e., slow walking) requires more cognitive control, which might weaken a low-level link between walking phase and eye-movements. The other possibility might be that the power of the current study is too low to detect such a walking phase modulation during slow walking. A stride cycle during slow walking (~1.1 s) took about two times longer than a stride cycle during normal walking (~0.6 s). Therefore, the number of steps cycles available for analysis was much lower during slow walking as compared to normal walking in a 76.5-s testing window. Although we tend to assume that 70 steps cycles should be enough to capture a walking phase modulation effect during slow walking (if there is one), further studies should explicitly test this possibility by extending the testing time.

### Limitations of the Current Study

Mobile EEG during unrestricted free walking always poses extra challenges due to its deviation from controlled stationary lab routines. We discuss these problems throughout the manuscript but want to shortly summarize and partly extend our considerations concerning artifacts and experimental limitations. In short, our main finding regarding the alpha power reduction due to walking was a frequency-band limited effect clearly excluding the problem of changed signal to noise ratio due to walking. Additionally, we controlled for eye movements, which are different between walking and standing still and pose a source of artifacts. We further measured impedance changes during walking with no indication that impedance changes could explain our findings.

Another challenge for mobile EEG is the reduced number of sensors. Our number of 24 sensors, including 6 sensors for EOG, certainly does not permit a further analysis in source space. Nevertheless, as investigated in detail in a study by Lau et al. (2012), the number of sensors necessary for recording predominant electro-cortical sources is not large. Indeed, particularly for occipital or temporal activity, their results suggest that the pattern do not exhibit large changes by reducing the sensor number from 125 to 25 [see Figure 4 (Lau et al., 2012)]. Therefore, staying in sensor space, ICA as applied in the current study is able to mark hotspots of activation, and the low number of sensors does not introduce a distortion but only results in a reduced spatial resolution. In our approach, we further picked those sources with a dominance over occipital area for further analysis. Importantly, it could have been feasible that even though we picked a focus over occipital sites, the difference between speed levels was introduced by changes with an only marginally overlapping spatial distribution. However, our Figure 1C (still – normal walking) clearly suggests that for the chosen components also the difference between speed levels was maximal at the power maximum. If the difference between speed levels was introduced by other sites, the difference should show a different distribution. Therefore, while the used number of sensor was suboptimal, our analysis and results nevertheless can demonstrate a change in power happening over occipital sites. Related to this, natural overground walking is inevitably associated with various sources of sensory input including auditory feedback, which might pose another possibility in explaining the alpha modulation during walking. In the current study, all participants walked on an indoor wooden floor with their own comfortable flat shoes. The auditory feedback from stepping was therefore weak but nevertheless clearly noticeable. However, an auditory explanation of the alpha modulation is highly unlikely for the following three reasons: 1. Auditory feedback from self actions typically show attenuated responses, which should lead to an even weaker neural response to auditory feedback during walking (Schneider et al., 2018); 2. We used ICA to restrict our analysis on alpha activities showing a distribution in the occipital area; 3. Auditory alpha is more difficult to detect than the visual alpha (Weisz et al., 2011). It is therefore unlikely that auditory alpha spilled majorly into occipital alpha sources.

The walking phase was estimated from the combined walking speed data collected from both ankles. While the low speed phase and high speed phase of a stride cycle can be obtained accurately, the exact moment of heel-strikes and toe-offs cannot be determined. Furthermore, the left foot stride and the right foot stride were not distinguished in the current study. Despite these differences from a canonical gait phase analysis (see e.g., Gwin et al., 2011), our results are consistent with previous findings with regards to the walking phase modulation of alpha power (Gwin et al., 2011; Chéron et al., 2012; Severens et al., 2012; Seeber et al., 2014, 2015; Bradford et al., 2016; Artoni et al., 2017; Oliveira et al., 2017). Therefore, the current results are very likely replicable with a canonical gait phase analysis.

## Conclusion

Our work indicates that the basis of visual perception, including neuronal processes and eye related movements, is influenced not only by the current demands of the visual input but also by the current movement state of the body. The above mentioned interactions between eye and other body movements as well as the modulation of neuronal activity due to body movements suggest a large-scale process to optimize perception during natural behavior. These findings highlight the importance of investigating perceptual processes in natural settings and considering body movements and their interactions when analyzing perception.

## Data Availability Statement

The datasets generated for this study can be found in online repositories. The names of the repository/repositories and accession number(s) can be found in the article/Supplementary Material.

## Ethics Statement

The studies involving human participants were reviewed and approved by The Ethics Committee of Department of Psychology, University Würzburg. The patients/participants provided their written informed consent to participate in this study.

## Author Contributions

LC implemented the testing protocol, collected the data, analyzed the data, and wrote the manuscript. XC collected the data, and provided suggestions for revising the manuscript. BH developed the experiment, led the project, and wrote the manuscript. All authors contributed to the article and approved the submitted version.

## Conflict of Interest

The authors declare that the research was conducted in the absence of any commercial or financial relationships that could be construed as a potential conflict of interest.

## References

[B1] Artoni F. Fanciullacci C. Bertolucci F. Panarese A. Makeig S. Micera S. (2017). Unidirectional brain to muscle connectivity reveals motor cortex control of leg muscles during stereotyped walking. Neuroimage 159, 403–416. 10.1016/j.neuroimage.2017.07.013 28782683 PMC6698582

[B2] Ayaz A. Saleem A. B. Scholvinck M. L. Carandini M. (2013). Locomotion controls spatial integration in mouse visual cortex. Curr. Biol. 23, 890–894. 10.1016/j.cub.2013.04.012 23664971 PMC3661981

[B3] Bauer L. O. Strock B. D. Goldstein R. Stern J. A. Walrath L. C. (1985). Auditory discrimination and the eyeblink. Psychophysiology 22, 636–641. 10.1111/j.1469-8986.1985.tb01660.x 4089089

[B4] Belyusar D. Snyder A. C. Frey H. -P. Harwood M. R. Wallman J. Foxe J. J. (2013). Oscillatory alpha-band suppression mechanisms during the rapid attentional shifts required to perform an anti-saccade task. Neuroimage 65, 395–407. 10.1016/j.neuroimage.2012.09.061 23041338 PMC4380346

[B5] Benjamin A. V. Wailes-Newson K. Ma-Wyatt A. Baker D. H. Wade A. R. (2018). The effect of locomotion on early visual contrast processing in humans. J. Neurosci. 38, 3050–3059. 10.1523/JNEUROSCI.1428-17.2017 29463642 PMC5864146

[B6] Ben-Simon E. Podlipsky I. Okon-Singer H. Gruberger M. Cvetkovic D. Intrator N.. (2013). The dark side of the alpha rhythm: fMRI evidence for induced alpha modulation during complete darkness. Eur. J. Neurosci. 37, 795–803. 10.1111/ejn.12083 23216771

[B7] Bradford J. C. Lukos J. R. Ferris D. P. (2016). Electrocortical activity distinguishes between uphill and level walking in humans. J. Neurophysiol. 115, 958–966. 10.1152/jn.00089.2015 26683062

[B8] Bullock T. Cecotti H. Giesbrecht B. (2015). Multiple stages of information processing are modulated during acute bouts of exercise. Neuroscience 307, 138–150. 10.1016/j.neuroscience.2015.08.046 26318337

[B9] Bullock T. Elliott J. C. Serences J. T. Giesbrecht B. (2017). Acute exercise modulates feature-selective responses in human cortex. J. Cogn. Neurosci. 29, 605–618. 10.1162/jocn_a_01082 27897672

[B10] Busse L. Cardin J. A. Chiappe M. E. Halassa M. M. Mcginley M. J. Yamashita T.. (2017). Sensation during active behaviors. J. Neurosci. 37, 10826–10834. 10.1523/JNEUROSCI.1828-17.2017 29118211 PMC5678015

[B11] Cao L. Händel B. (2019). Walking enhances peripheral visual processing in humans. PLoS Biol. 17:e3000511. 10.1371/journal.pbio.3000511 31603894 PMC6808500

[B12] Castermans T. Duvinage M. Cheron G. Dutoit T. (2014). About the cortical origin of the low-delta and high-gamma rhythms observed in EEG signals during treadmill walking. Neurosci. Lett. 561, 166–170. 10.1016/j.neulet.2013.12.059 24412128

[B13] Chéron G. Duvinage M. De Saedeleer C. Castermans T. Bengoetxea A. Petieau M.. (2012). From spinal central pattern generators to cortical network: integrated BCI for walking rehabilitation. Neural Plast. 2012:375148. 10.1155/2012/375148 22272380 PMC3261492

[B14] Chiappe M. E. Seelig J. D. Reiser M. B. Jayaraman V. (2010). Walking modulates speed sensitivity in Drosophila motion vision. Curr. Biol. 20, 1470–1475. 10.1016/j.cub.2010.06.072 20655222 PMC4435946

[B15] Cho P. Sheng C. Chan C. Lee R. Tam J. (2000). Baseline blink rates and the effect of visual task difficulty and position of gaze. Curr. Eye Res. 20, 64–70. 10.1076/0271-3683(200001)2011-HFT064 10611717

[B16] Clancy K. B. Orsolic I. Mrsic-Flogel T. D. (2019). Locomotion-dependent remapping of distributed cortical networks. Nat. Neurosci. 22, 778–786. 10.1038/s41593-019-0357-8 30858604 PMC6701985

[B17] Collewijn H. Kowler E. (2008). The significance of microsaccades for vision and oculomotor control. J. Vis. 8, 20.1–21. 10.1167/8.14.20 19146321 PMC3522523

[B18] Cong D. K. Sharikadze M. Staude G. Deubel H. Wolf W. (2010). Spontaneous eye blinks are entrained by finger tapping. Hum. Mov. Sci. 29, 1–18. 10.1016/j.humov.2009.08.003 19913931

[B19] Conradi N. Abel C. Frisch S. Kell C. A. Kaiser J. Schmidt-Kassow M. (2016). Actively but not passively synchronized motor activity amplifies predictive timing. Neuroimage 139, 211–217. 10.1016/j.neuroimage.2016.06.033 27329809

[B20] Cram J. R. Kohlenberg R. J. Singer M. (1977). Operant control of alpha EEG and the effects of illumination and eye closure. Psychosom. Med. 39, 11–18. 10.1097/00006842-197701000-00002 847075

[B21] Cruz A. A. Garcia D. M. Pinto C. T. Cechetti S. P. (2011). Spontaneous eyeblink activity. Ocul. Surf. 9, 29–41. 10.1016/S1542-0124(11)70007-6 21338567

[B22] Dadarlat M. C. Stryker M. P. (2017). Locomotion enhances neural encoding of visual stimuli in mouse V1. J. Neurosci. 37, 3764–3775. 10.1523/JNEUROSCI.2728-16.2017 28264980 PMC5394894

[B23] De Sanctis P. Butler J. S. Malcolm B. R. Foxe J. J. (2014). Recalibration of inhibitory control systems during walking-related dual-task interference: a mobile brain-body imaging (MOBI) study. Neuroimage 94, 55–64. 10.1016/j.neuroimage.2014.03.016 24642283 PMC4209901

[B24] De Vos M. Gandras K. Debener S. (2014). Towards a truly mobile auditory brain-computer interface: exploring the P300 to take away. Int. J. Psychophysiol. 91, 46–53. 10.1016/j.ijpsycho.2013.08.010 23994208

[B25] Delorme A. Makeig S. (2004). EEGLAB: an open source toolbox for analysis of single-trial EEG dynamics including independent component analysis. J. Neurosci. Methods. 134, 9–21. 10.1016/j.jneumeth.2003.10.009 15102499

[B26] Dipoppa M. Ranson A. Krumin M. Pachitariu M. Carandini M. Harris K. D. (2018). Vision and locomotion shape the interactions between neuron types in mouse visual cortex. Neuron 98, 602–615. e608. 10.1016/j.neuron.2018.03.037 29656873 PMC5946730

[B27] Ehinger B. V. Fischer P. Gert A. L. Kaufhold L. Weber F. Pipa G.. (2014). Kinesthetic and vestibular information modulate alpha activity during spatial navigation: a mobile EEG study. Front. Hum. Neurosci. 8:71. 10.3389/fnhum.2014.00071 24616681 PMC3934489

[B28] Erisken S. Vaiceliunaite A. Jurjut O. Fiorini M. Katzner S. Busse L. (2014). Effects of locomotion extend throughout the mouse early visual system. Curr. Biol. 24, 2899–2907. 10.1016/j.cub.2014.10.045 25484299

[B29] Ferree T. C. Luu P. Russell G. S. Tucker D. M. (2001). Scalp electrode impedance, infection risk, and EEG data quality. Clin. Neurophysiol. 112, 536–544. 10.1016/S1388-2457(00)00533-2 11222977

[B30] Fu K. M. Foxe J. J. Murray M. M. Higgins B. A. Javitt D. C. Schroeder C. E. (2001). Attention-dependent suppression of distracter visual input can be cross-modally cued as indexed by anticipatory parieto-occipital alpha-band oscillations. Brain Res. Cogn. Brain Res. 12, 145–152. 10.1016/S0926-6410(01)00034-9 11489617

[B31] Fu Y. Tucciarone J. M. Espinosa J. S. Sheng N. Darcy D. P. Nicoll R. A.. (2014). A cortical circuit for gain control by behavioral state. Cell 156, 1139–1152. 10.1016/j.cell.2014.01.050 24630718 PMC4041382

[B32] García-Cossio E. Severens M. Nienhuis B. Duysens J. Desain P. Keijsers N.. (2015). Decoding sensorimotor rhythms during robotic-assisted treadmill walking for brain computer interface (BCI) applications. PLoS ONE 10:e0137910. 10.1371/journal.pone.0137910 26675472 PMC4686050

[B33] Gladwin T. E. (2020). An implementation of N-way repeated measures ANOVA: effect coding, automated unpacking of interactions, and randomization testing. MethodsX 7:100947. 10.1016/j.mex.2020.100947 32612937 PMC7315100

[B34] Gramann K. Gwin J. T. Bigdely-Shamlo N. Ferris D. P. Makeig S. (2010). Visual evoked responses during standing and walking. Front. Hum. Neurosci. 4:202. 10.3389/fnhum.2010.00202 21267424 PMC3024562

[B35] Gwin J. T. Gramann K. Makeig S. Ferris D. P. (2011). Electrocortical activity is coupled to gait cycle phase during treadmill walking. Neuroimage 54, 1289–1296. 10.1016/j.neuroimage.2010.08.066 20832484

[B36] Haegens S. Nacher V. Luna R. Romo R. Jensen O. (2011). alpha-Oscillations in the monkey sensorimotor network influence discrimination performance by rhythmical inhibition of neuronal spiking. Proc. Natl. Acad. Sci. U. S. A. 108, 19377–19382. 10.1073/pnas.1117190108 22084106 PMC3228466

[B37] Händel B. F. Haarmeier T. Jensen O. (2011). Alpha oscillations correlate with the successful inhibition of unattended stimuli. J. Cogn. Neurosci. 23, 2494–2502. 10.1162/jocn.2010.21557 20681750

[B38] Händel B. F. Scholvinck M. L. (2017). The brain during free movement - What can we learn from the animal model. Brain Res. 1716, 3–15. 10.1016/j.brainres.2017.09.003 28893579

[B39] Hollands M. A. Marple-Horvat D. E. (2001). Coordination of eye and leg movements during visually guided stepping. J. Mot. Behav. 33, 205–216. 10.1080/00222890109603151 11404215

[B40] Keller G. B. Bonhoeffer T. Hubener M. (2012). Sensorimotor mismatch signals in primary visual cortex of the behaving mouse. Neuron 74, 809–815. 10.1016/j.neuron.2012.03.040 22681686

[B41] Kelly S. P. Lalor E. C. Reilly R. B. Foxe J. J. (2006). Increases in alpha oscillatory power reflect an active retinotopic mechanism for distracter suppression during sustained visuospatial attention. J. Neurophysiol. 95, 3844–3851. 10.1152/jn.01234.2005 16571739

[B42] Keren A. S. Yuval-Greenberg S. Deouell L. Y. (2010). Saccadic spike potentials in gamma-band EEG: characterization, detection and suppression. Neuroimage 49, 2248–2263. 10.1016/j.neuroimage.2009.10.057 19874901

[B43] Klimesch W. (1999). EEG alpha and theta oscillations reflect cognitive and memory performance: a review and analysis. Brain Res. Brain Res. Rev. 29, 169–195. 10.1016/S0165-0173(98)00056-3 10209231

[B44] Klimesch W. Sauseng P. Hanslmayr S. (2007). EEG alpha oscillations: the inhibition-timing hypothesis. Brain Res. Rev. 53, 63–88. 10.1016/j.brainresrev.2006.06.003 16887192

[B45] Kowler E. (1991). “The role of visual and cognitive processes in the control of eye movement,” in Eye Movements and Their Role in Visual and Cognitive Processes, ed E. Kowler (Amsterdam: Elsevier), 1–70. 7492527

[B46] Kranczioch C. Zich C. Schierholz I. Sterr A. (2014). Mobile EEG and its potential to promote the theory and application of imagery-based motor rehabilitation. Int. J. Psychophysiol. 91, 10–15. 10.1016/j.ijpsycho.2013.10.004 24144637

[B47] Labonté-Lemoyne É. Santhanam R. Léger P.-M. Courtemanche F. Fredette M. Sénécal S. (2015). The delayed effect of treadmill desk usage on recall and attention. Comp. Hum. Behav. 46, 1–5. 10.1016/j.chb.2014.12.054

[B48] Lau T. M. Gwin J. T. Ferris D. P. (2012). How many electrodes are really needed for EEG-based mobile brain imaging? J. Behav. Brain Sci. 2, 387–393. 10.4236/jbbs.2012.23044

[B49] Lee A. M. Hoy J. L. Bonci A. Wilbrecht L. Stryker M. P. Niell C. M. (2014). Identification of a brainstem circuit regulating visual cortical state in parallel with locomotion. Neuron 83, 455–466. 10.1016/j.neuron.2014.06.031 25033185 PMC4151326

[B50] Lin Y. P. Wang Y. Wei C. S. Jung T. P. (2014). Assessing the quality of steady-state visual-evoked potentials for moving humans using a mobile electroencephalogram headset. Front. Hum. Neurosci. 8:182. 10.3389/fnhum.2014.00182 24744718 PMC3978365

[B51] Maimon G. Straw A. D. Dickinson M. H. (2010). Active flight increases the gain of visual motion processing in Drosophila. Nat. Neurosci. 13, 393–399. 10.1038/nn.2492 20154683

[B52] Matin E. (1974). Saccadic suppression: a review and an analysis. Psychol. Bull. 81, 899–917. 10.1037/h0037368 4612577

[B53] Matthis J. S. Barton S. L. Fajen B. R. (2017). The critical phase for visual control of human walking over complex terrain. Proc. Natl. Acad. Sci. U. S. A. 114, E6720–E6729. 10.1073/pnas.1611699114 28739912 PMC5558990

[B54] Matthis J. S. Yates J. L. Hayhoe M. M. (2018). Gaze and the control of foot placement when walking in natural terrain. Curr. Biol. 28, 1224–1233. e1225. 10.1016/j.cub.2018.03.008 29657116 PMC5937949

[B55] Mcmorris T. Graydon J. (2000). The effect of incremental exercise on cognitive performance. Int. J. Sport Psychol. 31, 66–81.

[B56] Nathan K. Contreras-Vidal J. L. (2015). Negligible motion artifacts in scalp electroencephalography (EEG) during treadmill walking. Front. Hum. Neurosci. 9:708. 10.3389/fnhum.2015.00708 26793089 PMC4710850

[B57] Niell C. M. Stryker M. P. (2010). Modulation of visual responses by behavioral state in mouse visual cortex. Neuron 65, 472–479. 10.1016/j.neuron.2010.01.033 20188652 PMC3184003

[B58] Oliveira A. S. Schlink B. R. Hairston W. D. Konig P. Ferris D. P. (2017). Restricted vision increases sensorimotor cortex involvement in human walking. J. Neurophysiol. 118, 1943–1951. 10.1152/jn.00926.2016 28679843 PMC5626901

[B59] Oostenveld R. Fries P. Maris E. Schoffelen J. M. (2011). FieldTrip: open source software for advanced analysis of MEG, EEG, and invasive electrophysiological data. Comput. Intell. Neurosci. 2011:156869. 10.1155/2011/156869 21253357 PMC3021840

[B60] Polack P.-O. Friedman J. Golshani P. (2013). Cellular mechanisms of brain state-dependent gain modulation in visual cortex. Nat. Neurosci. 16, 1331–1339. 10.1038/nn.3464 23872595 PMC3786578

[B61] Roeder L. Boonstra T. W. Smith S. S. Kerr G. K. (2018). Dynamics of corticospinal motor control during overground and treadmill walking in humans. J. Neurophysiol. 120, 1017–1031. 10.1152/jn.00613.2017 29847229

[B62] Saleem A. B. Ayaz A. Jeffery K. J. Harris K. D. Carandini M. (2013). Integration of visual motion and locomotion in mouse visual cortex. Nat. Neurosci. 16, 1864–1869. 10.1038/nn.3567 24185423 PMC3926520

[B63] Sauseng P. Klimesch W. Stadler W. Schabus M. Doppelmayr M. Hanslmayr S.. (2005). A shift of visual spatial attention is selectively associated with human EEG alpha activity. Eur. J. Neurosci. 22, 2917–2926. 10.1111/j.1460-9568.2005.04482.x 16324126

[B64] Scanlon J. E.M. Townsend K. A. Cormier D. L. Kuziek J. W.P. Mathewson K. E. (2017). Taking off the training wheels: measuring auditory P3 during outdoor cycling using an active wet EEG system. Brain Res. 1716, 50–61. 10.1101/157941 29248602

[B65] Schmidt-Kassow M. Heinemann L. V. Abel C. Kaiser J. (2013). Auditory–motor synchronization facilitates attention allocation. Neuroimage 82, 101–106. 10.1016/j.neuroimage.2013.05.111 23732882

[B66] Schneider D. M. Sundararajan J. Mooney R. (2018). A cortical filter that learns to suppress the acoustic consequences of movement. Nature 561, 391–395. 10.1038/s41586-018-0520-5 30209396 PMC6203933

[B67] Seeber M. Scherer R. Wagner J. Solis-Escalante T. Müller-Putz G. R. (2014). EEG beta suppression and low gamma modulation are different elements of human upright walking. Front. Hum. Neurosci. 8:485. 10.3389/fnhum.2014.00485 25071515 PMC4086296

[B68] Seeber M. Scherer R. Wagner J. Solis-Escalante T. Müller-Putz G. R. (2015). High and low gamma EEG oscillations in central sensorimotor areas are conversely modulated during the human gait cycle. Neuroimage 112, 318–326. 10.1016/j.neuroimage.2015.03.045 25818687

[B69] Severens M. Nienhuis B. Desain P. Duysens J. (2012). “Feasibility of measuring event related desynchronization with electroencephalography during walking,” in: 2012 Annual International Conference of the IEEE Engineering in Medicine and Biology Society: IEEE, 2764–2767. 23366498 10.1109/EMBC.2012.6346537

[B70] Snyder K. L. Kline J. E. Huang H. J. Ferris D. P. (2015). Independent component analysis of gait-related movement artifact recorded using EEG electrodes during treadmill walking. Front. Hum. Neurosci. 9:639. 10.3389/fnhum.2015.00639 26648858 PMC4664645

[B71] Storzer L. Butz M. Hirschmann J. Abbasi O. Gratkowski M. Saupe D.. (2016). Bicycling and walking are associated with different cortical oscillatory dynamics. Front. Hum Neurosci. 10:61. 10.3389/fnhum.2016.00061 26924977 PMC4759288

[B72] Thut G. Nietzel A. Brandt S. A. Pascual-Leone A. (2006). Alpha-band electroencephalographic activity over occipital cortex indexes visuospatial attention bias and predicts visual target detection. J. Neurosci. 26, 9494–9502. 10.1523/JNEUROSCI.0875-06.2006 16971533 PMC6674607

[B73] Van Dam L. C.J. Van Ee R. (2005). The role of (micro) saccades and blinks in perceptual bi-stability from slant rivalry. Vis. Res. 45, 2417–2435. 10.1016/j.visres.2005.03.013 15894347

[B74] Van Dijk H. Schoffelen J. M. Oostenveld R. Jensen O. (2008). Prestimulus oscillatory activity in the alpha band predicts visual discrimination ability. J. Neurosci. 28, 1816–1823. 10.1523/JNEUROSCI.1853-07.2008 18287498 PMC6671447

[B75] Vinck M. Batista-Brito R. Knoblich U. Cardin J. A. (2015). Arousal and locomotion make distinct contributions to cortical activity patterns and visual encoding. Neuron 86, 740–754. 10.1016/j.neuron.2015.03.028 25892300 PMC4425590

[B76] Volkmann F. C. Riggs L. A. Moore R. K. (1980). Eyeblinks and visual suppression. Science 207, 900–902. 10.1126/science.7355270 7355270

[B77] Von Cramon D. Schuri U. (1980). Blink frequency and speed motor activity. Neuropsychologia 18, 603–606. 10.1016/0028-3932(80)90164-5 7443026

[B78] Wagner J. Solis-Escalante T. Grieshofer P. Neuper C. Müller-Putz G. Scherer R. (2012). Level of participation in robotic-assisted treadmill walking modulates midline sensorimotor EEG rhythms in able-bodied subjects. Neuroimage 63, 1203–1211. 10.1016/j.neuroimage.2012.08.019 22906791

[B79] Wagner J. Solis-Escalante T. Scherer R. Neuper C. Müller-Putz G. (2014). It's how you get there: walking down a virtual alley activates premotor and parietal areas. Front. Hum. Neurosci. 8:93. 10.3389/fnhum.2014.00093 24611043 PMC3933811

[B80] Wascher E. Heppner H. Hoffmann S. (2014). Towards the measurement of event-related EEG activity in real-life working environments. Int. J. Psychophysiol. 91, 3–9. 10.1016/j.ijpsycho.2013.10.006 24144635

[B81] Weir P. T. Schnell B. Dickinson M. H. (2014). Central complex neurons exhibit behaviorally gated responses to visual motion in Drosophila. J. Neurophysiol. 111, 62–71. 10.1152/jn.00593.2013 24108792

[B82] Weisz N. Hartmann T. Müller N. Obleser J. (2011). Alpha rhythms in audition: cognitive and clinical perspectives. Front. Psychol. 2:73. 10.3389/fpsyg.2011.00073 21687444 PMC3110491

[B83] Wilcox R. R. Rousselet G. A. (2018). A guide to robust statistical methods in neuroscience. Curr. Protoc. Neurosci. 82, 8:42.1–48:42.30. 10.1002/cpns.41 29357109

[B84] Worden M. S. Foxe J. J. Wang N. Simpson G. V. (2000). Anticipatory biasing of visuospatial attention indexed by retinotopically specific alpha-band electroencephalography increases over occipital cortex. J Neurosci. 20:RC63. 10.1523/JNEUROSCI.20-06-j0002.2000 10704517 PMC6772495

[B85] Yamagishi N. Callan D. E. Goda N. Anderson S. J. Yoshida Y. Kawato M. (2003). Attentional modulation of oscillatory activity in human visual cortex. Neuroimage 20, 98–113. 10.1016/S1053-8119(03)00341-0 14527573

[B86] Yekutieli D. Benjamini Y. (1999). Resampling-based false discovery rate controlling multiple test procedures for correlated test statistics. J. Stat. Plan. Inference 82, 171–196. 10.1016/S0378-3758(99)00041-5

