# Supplementary material for: Overground Walking Decreases Alpha Activity and Entrains Eye Movements in Humans
Source: Front Hum Neurosci. 2020 Dec 22;14:561755. doi: 10.3389/fnhum.2020.561755 (PMC7782973; doi:10.3389/fnhum.2020.561755)
Supplement: Supplementary file 1 [file Data_Sheet_1.pdf]

Supplementary materials for

Overground walking decreases alpha activity and entrains eye movements in humans

Liyu Cao, Xinyu Chen, & Barbara Haendel

Supplementary Figure S1-S3

A Saccadic spike potentials

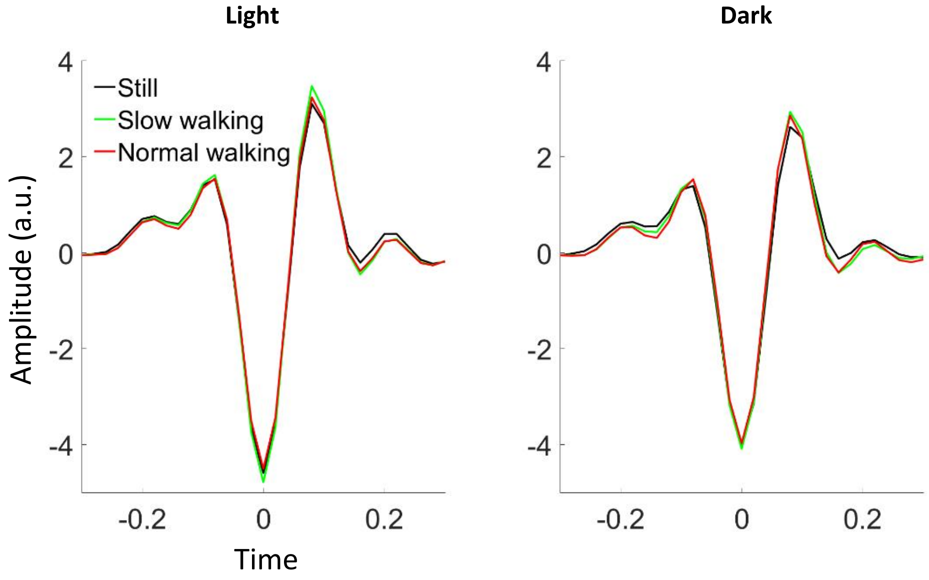

B Pupil size standard deviation

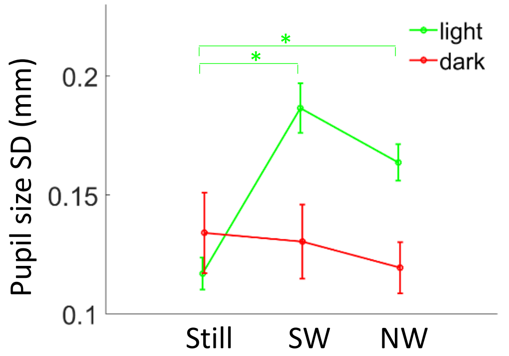

C Amplitude of saccadic spike potential across walking phase

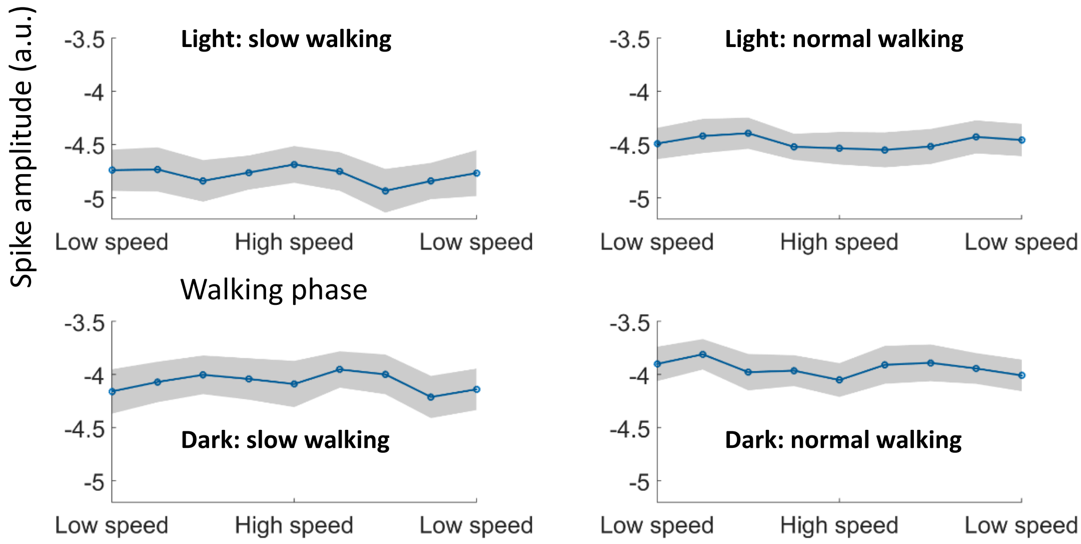

Supplementary Figure S1. Saccadic spike potentials and pupil size variance. (**A**) Group average saccadic spike potentials in each testing session. For each participant, each detected saccade was aligned to the lowest amplitude point (time 0) and referenced to the mean amplitude of the Hilbert transformed REOG signal within each testing session through division. Clear saccadic spike potentials indicate reliable detection of saccades. See methods section for details of the saccade detection method. (**B**) The standard deviation of pupil size in each condition. A 2 (lighting condition: light vs. dark) by 3 (speed condition: standing still, slow walking, and normal walking) within-subjects ANOVA led to significant main effects of lighting ($F(1,27) = 4.66$, $p = 0.04$) and speed ($F(2,54) = 6.05$, $p = 0.007$), as well as a significant interaction effect ($F(2,54) = 7.89$, $p < 0.001$). Post-hoc analysis showed that the pupil size standard deviation was higher during walking than during standing in the light (slow walking vs. standing still: ($t(27) = 7.64$, $p < 0.001$; normal walking vs. standing still: ($t(27) = 4.53$, $p < 0.001$; normal walking vs. slow walking: ($t(27) = -1.96$, $p = 0.07$), whereas no such difference was found in the dark (all *p*s > 0.39). Asterisks indicate $p < 0.05$. Vertical lines indicate ±1 standard error. N = 28. SW: slow walking; NW: normal walking. (**C**) Walking phase does not modulate the amplitude of saccadic spike potentials. For each detected saccade, the amplitude of saccadic spike potential was taken at the time point of 0. A 2 (lighting condition: light vs. dark) by 2 (walking speed condition: slow vs. normal walking) by 9 (walking phase) within-subjects ANOVA was performed to test the amplitude. Significant main effects were found for lighting ($F(1,28) = 39.26$, $p < 0.001$) and walking speed ($F(1,28) = 8.50$, $p = 0.01$). Note that the threshold for saccade detection was determined separately for each testing session. Therefore, the amplitude difference between testing sessions would not contribute to the difference in the number of saccades deteted between sessions. No other effects for the ANOVA were significant. Shading indicates ±1 standard error. N = 29.

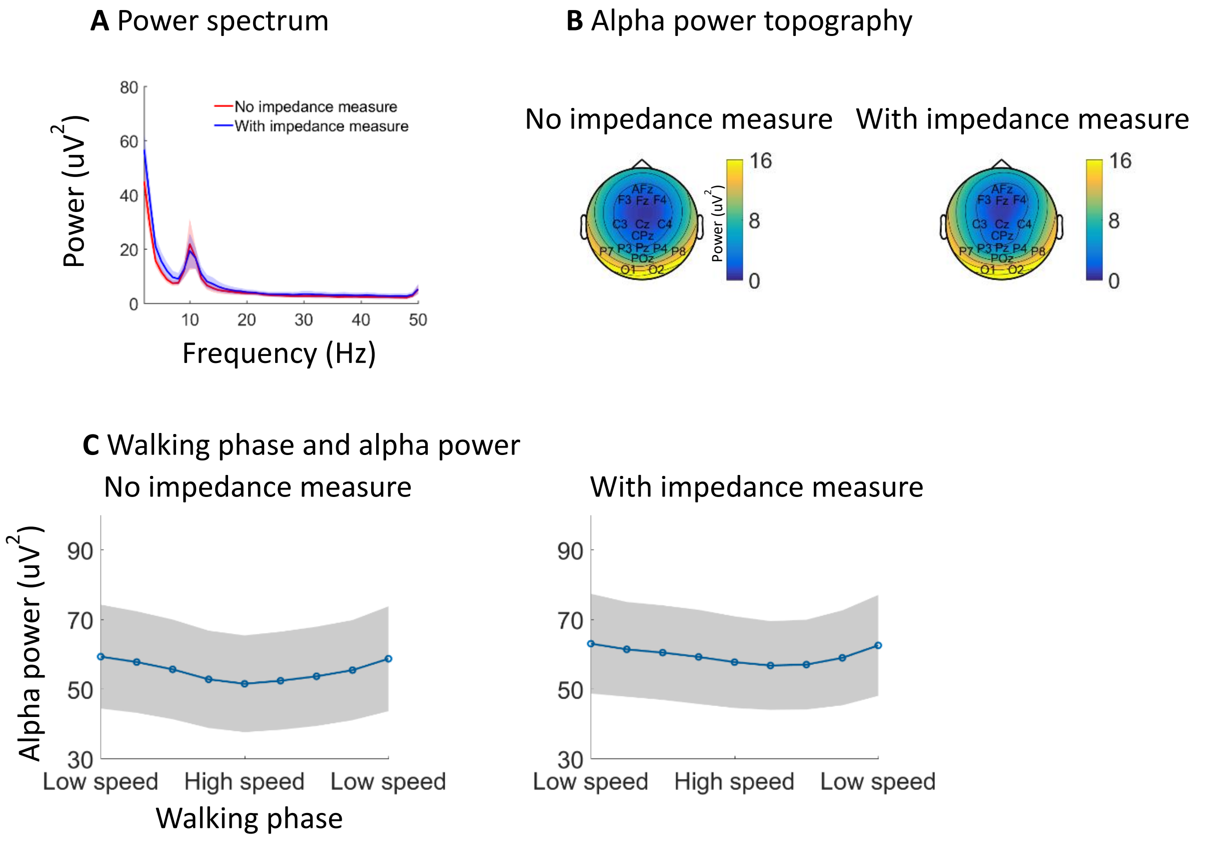

Supplementary Figure S2. Impedance measure dataset. (**A**) The average EEG power spectra from four electrodes (‘O1’, ’O2’, ’P7’, ’P8’), with the impedance measure switched on or switched off. No significant influence was found on the power spectrum from the impedance measure. Shading indicates ±1 standard error. N = 18. (**B**) The topography of alpha power. (**C**) Alpha power in different walking phases. The alpha power was averaged over the four electrodes whose power spectra were plotted in (**A**). A 2 (impedance measure: no vs. yes) by 9 (walking phase) within-subjects ANOVA showed a significant main effect of walking phase (F(8,120) = 4.72, *p* = 0.01), which is similar to the results obtained with ICA components (Fig. 3B in the main text). No other effects were significant (impedance measure: F(1,15) = 0.17, *p* = 0.66; interaction: F(8,120) = 0.91, *p* = 0.49). Shading indicates ±1 standard error. N = 16.

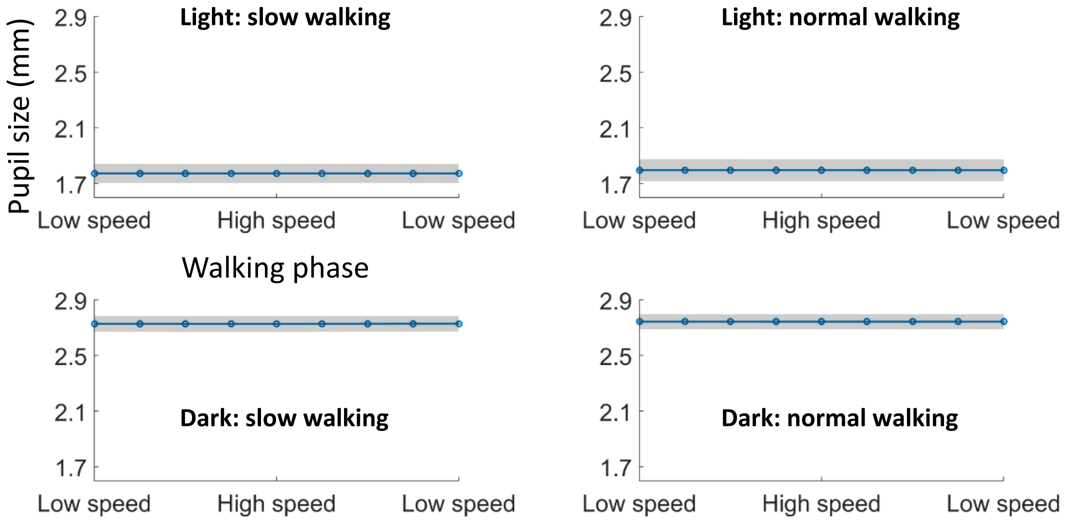

Supplementary Figure S3. Walking phase does not modulate pupil size. The pupil size did not show any difference in different walking phases. Shading indicates ±1 standard error. N = 27.
